# Coding deficits in hidden hearing loss induced by noise: the nature and impacts

**DOI:** 10.1038/srep25200

**Published:** 2016-04-27

**Authors:** Qiang Song, Pei Shen, Xiaowei Li, Lijuan Shi, Lijie Liu, Jiping Wang, Zhiping Yu, Kegan Stephen, Steve Aiken, Shankai Yin, Jian Wang

**Affiliations:** 1 Department of Otolaryngology, Affiliated Sixth People’s Hospital, Shanghai Jiao Tong University, 600 Yishan Road, Shanghai 200233, China; 2 Department of Physiology, Medical College of Southeast University, 87 Dingjiaoqiao Road, Nanjing 210009, China; 3 School of Human Communication Disorders, Dalhousie University, 1256 Barrington St. Dalhousie University, Halifax, NS B3J1Y6, Canada

## Abstract

Hidden hearing refers to the functional deficits in hearing without deterioration in hearing sensitivity. This concept is proposed based upon recent finding of massive noise-induced damage on ribbon synapse between inner hair cells (IHCs) and spiral ganglion neurons (SGNs) in the cochlea without significant permanent threshold shifts (PTS). Presumably, such damage may cause coding deficits in auditory nerve fibers (ANFs). However, such deficits had not been detailed except that a selective loss of ANFs with low spontaneous rate (SR) was reported. In the present study, we investigated the dynamic changes of ribbon synapses and the coding function of ANF single units in one month after a brief noise exposure that caused a massive damage of ribbon synapses but no PTS. The synapse count and functional response measures indicates a large portion of the disrupted synapses were re-connected. This is consistent with the fact that the change of SR distribution due to the initial loss of low SR units is recovered quickly. However, ANF coding deficits were developed later with the re-establishment of the synapses. The deficits were found in both intensity and temporal processing, revealing the nature of synaptopathy in hidden hearing loss.

The ribbon synapse between spiral ganglion neurons (SGNs) and cochlear hair cells (HCs), particularly inner hair cells (IHCs), has been found as a new locus of noise induced cochlear damage. In adult rodents (both in mice and guinea pigs), a brief noise exposure at a relatively low dose may not cause permanent threshold shift (PTS) of hearing but produce massive damage on the ribbon synapses, resulting the disconnection of may auditory nerve fibers (ANFs) with their targeted IHCs^1^,^2^,^3^,^4^. Without a PTS, such damage should not be aware of by human subjects and will be missed by routine audiology evaluations, which are focused mainly on hearing threshold. Therefore, this kind of acoustic trauma is silent or subclinical. However, the damage on ribbon synapse reduces the cochlear output to auditory brain and probably changes other aspects of the cochlear signal processing, especially temporal coding^3^,^4^. To count for the functional deficits in hearing that may be resulted from the cochlear damage without a threshold change (such as seen after a noise exposure without PTS), a concept hidden hearing loss has been proposed^5^.

The nature and the functional significance of hidden hearing loss need to be comprehensively evaluated because noise exposure causing such hearing loss occurs frequently in our daily life and is often considered as safe according to current standard. Recently, a single unit study demonstrated that a no-PTS noise exposure selectively damaged the synapses with auditory nerve fibers (ANF) that were low in spontaneous rate (SR) of action potentials^6^. This finding is important because the low SR ANF units have relatively larger dynamic range and wide threshold distribution^7^,^8^. Therefore, those units are more important in signal coding in noisy background where those high SR units are saturated by the noise due to their narrow dynamic range and low thresholds^5^. Since the observation is at one time point after the noise exposure, we do not know if the loss of low SR units is permanent or can be repaired. Surprisingly, no changes in ANF coding functions were verified in this report after a massive loss of low SR ANFs^6^. Therefore, we virtually know nothing about the nature of the hidden hearing loss induced by a no-PTS noise exposure so far, other than the prediction from the loss of low SR units. In our previous reports, we have observed the changes in auditory evoked field responses and the number of ribbon-synapse at multiple time points after such a no-PTS noise exposure in guinea pigs. We found that the number of synapses (verified in immunohistology staining against presynaptic ribbons and postsynaptic terminals) are largely recovered following a massive initial reduction^3^,^4^. However, functional deterioration in signal processing, especially the temporal processing ability appeared at later phase (towards one month after the noise) when the ribbon synapse numbers were largely recovered. This result suggested that the repaired synapses are functionally abnormal.

In the present study, we investigated the dynamic changes of coding activities of single ANF units after a no-PTS noise exposure that was similar to what was utilized in in our previous studies^3^,^4^. The single unit recording was combined with other functional observation and the changes in the ribbon synapse morphology observed under transmission electronics microscope (TEM). Evidence collected are consistent with the idea that the damaged synapses are partially and largely repairable, and because of the repair, the change of SR distribution by the initial loss of low-SR units is transient and recovered later. Most significantly, the repaired synapses are functionally abnormal in both intensity and temporal coding.

## Results

Similar to our previous reports^3^,^4^, a brief exposure to a broadband noise at 105 dB SPL produced a significant temporal threshold shift (TTS) of moderate degree as tested in the audiogram of auditory brainstem responses (ABR) at 1 day post noise (1DPN); however, no significant difference in ABR thresholds was seen between the control and those tested at 1 week and 1 month post noise (1WPN and 1MPN, Supplementary Fig. 1), suggesting that the noise exposure did not cause a PTS. In addition to the total recovery of ABR threshold, there was a total recovery in distortion product of otoacoustic emission (DPOAE, sFig. 2), suggesting a virtually total recovery of outer hair cell (OHC) function.

**Figure 1:**
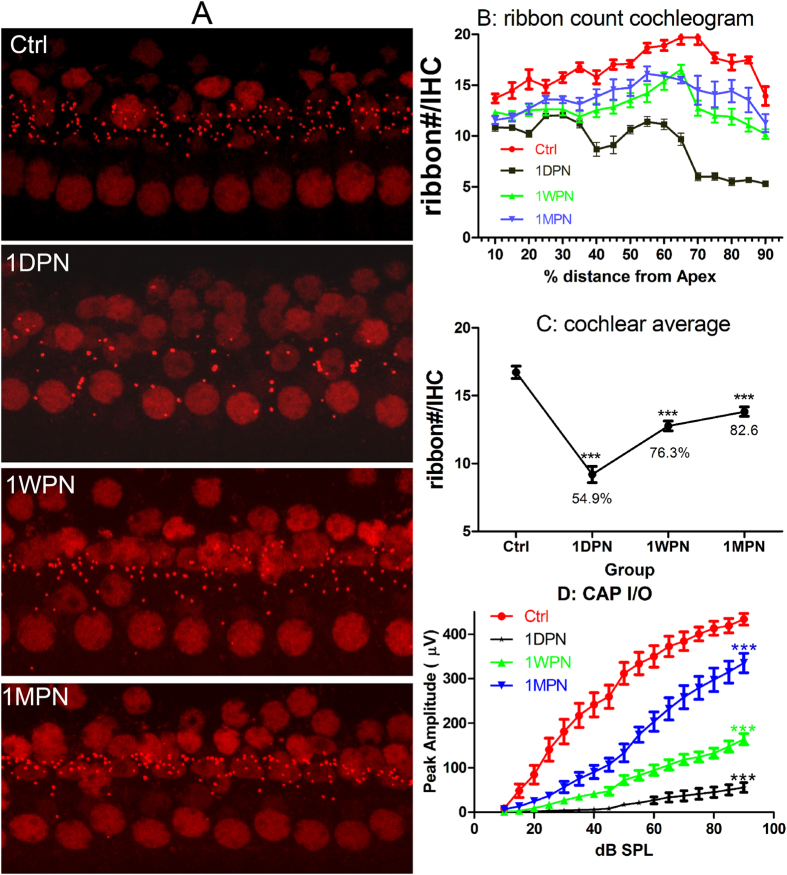
Noise induced changes in ribbon density (ribbon#/IHC) and click evoked CAP amplitude. (**A**) representative images showing ribbon changes by the noise exposure. (**B**) ribbon#/IHC cochleogram. (**C**) the changes of overall ribbon density after noise (% was calculated against the value of control). (**D**) Click-evoked CAP input-output functions. A one-way ANOVAs were performed on the overall ribbon density changes and the CAP amplitude measured at 90 dB SPL. Both showed significant effects of noise. The significance was indicated by the asterisks as the result of post-hoc pairwise tests against the control. ***p < 0.001.

Figure 1 summarized the impact of the noise exposure on the number of ribbon synapses and the compound action potential (CAP). The number of ribbon synapses was represented by the count of ribbons stained with antibody against a ribbon protein: C-terminal binding protein 2 (CtBP2, Fig. 1A). Similar to what was reported in our previous studies^3^,^4^, an obvious reduction in ribbon number was seen at 1DPN, which was followed by a partial recovery at 1WPN and 1MPN. The ribbon numbers were counted successfully from 12–14 subjects (15–17 cochleae) in each group. In each cochlea, the count was done at 17 spots that were evenly distributed across the cochlea (Fig. 1B). At each spot, the ribbon count was averaged from 10–30 IHCs to generate ribbon density (the number of ribbon per IHC). The whole cochlea average of ribbon density was calculated thereafter. A reduction in the ribbon density was seen across the whole cochlear region, but more in the high frequency region. Figure 1C presents the cross-group comparison in the ribbon density calculated over the whole cochlea. The averaged ribbon number for each IHC was 16.73 ± 0.45 in the control group. The value was reduced to 9.191 ± 0.60 at 1DPN (reduced to 54.9% of the control). A partial recovery was seen later at 1WPN (12.77 ± 0.37, 76.3% of the control) and 1MPN (13.82 ± 0.35, 82.6 of the control). A one-way ANOVA was performed against the cochlear average of ribbon counts across groups. The result showed a significant effect of the noise treatment (F_3_ = 46.86, p < 0.0001). Post-hoc pairwise comparisons (Bonferroni tests) showed that the ribbon density of every post noise group was significantly lower than the control (t = 11.72, 6.16, 4.52 respectively for 1DPN, 1WPN and 1MPN versus the control, p < 0.001). Furthermore, the significant recovery from 1DPN was supported by the significance in the comparisons between 1DPN and 1WPN (t = 5.56, p < 0.001).

Click evoked CAP was recorded from 10–12 subjects in each group. Corresponding to the reduction in ribbon counts, the CAP amplitude was also reduced in all noise groups (Fig. 1D). A huge decrease of CAP was seen at 1DPN, followed by an incomplete recovery later. For example, at the highest click level tested (90 dB SPL), the CAP amplitude was 433.60 ± 12.89 μV in the control. This was dropped to 55.68 ± 10.96 335.56 (~12.8% of the control value) at 1DPN, much larger than the loss of ribbon density (Fig. 1B), suggesting an impaired function of ANFs connected with IHCs via survived synapses. At 1MPN, the CAP amplitude at the same click level was recovered to 335.56 ± 21.78 μV (77.4% of the control value). The reduction (23.6%) was still larger than the loss of ribbon count at this time point (17.4%, Fig. 1B), but much smaller than the initial loss of ribbons (45.1%) at 1DPN. Therefore, it is likely that some of the disrupted synapses are re-established. A significant effect of noise exposure was seen in a one-way ANOVA (F_3_ = 128.9, p < 0.001) on the CAP amplitude measured at this sound level. Compared with the control value, the CAP amplitude in each of the noise-exposed group was significantly lower than the control (post-hoc tests (Holm-Sidak method), t = 17.9, 12.3 and 12.7 for 1DPN, 1WPN and 1MPN respectively; p < 0.001 as indicated by the asterisks in Fig. 1C).

### Changes in Single Unit Activities

Approximately 200 single units of ANFs were recorded in each of the control and three noise-groups. After each unit was isolated by broadband noise bursts, the threshold of the unit was estimated using the noise bursts of different level. Then, the best frequency (BF) of the unit was determined in an iso-intensity curve tested 10 dB above the threshold estimated with noise-bursts.

**Figure 2:**
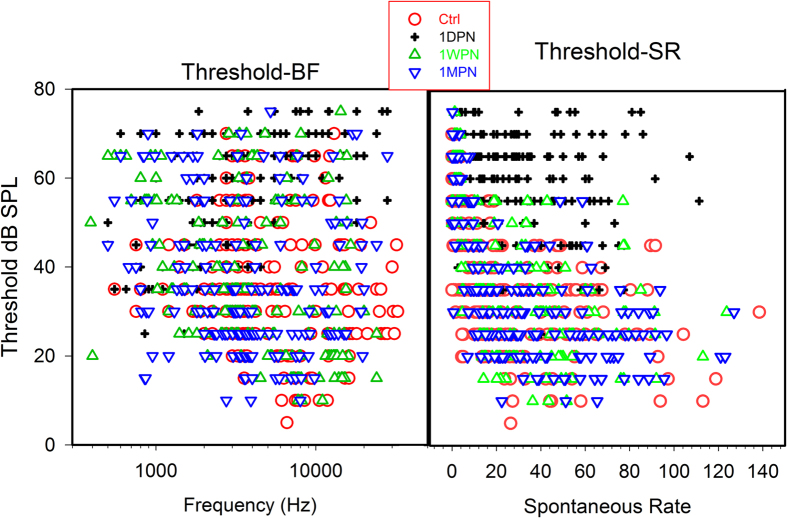
Distribution of threshold-BF (left) and threshold-SR (right) for all the units recorded from the four groups. The impact of the threshold elevation was seen at 1DPN. At this time, there was a disproportional loss of units with extremely low SR.

**Figure 3:**
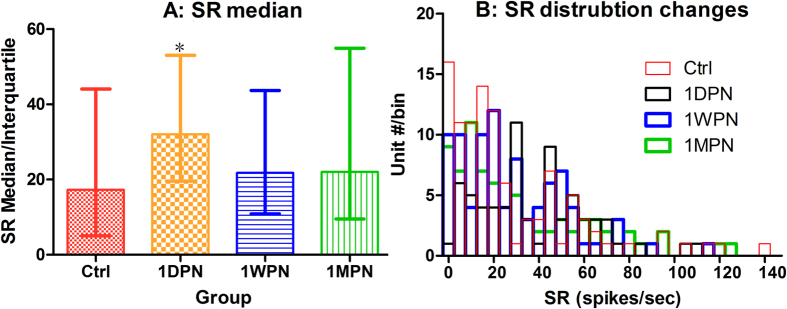
Noise-induced SR changes in units with BF >4kHz. (**A**) The changes in SR median. (**B**) The changes in SR distribution. One-way ANOVA of rank shows that a significant increase in SR exists only for those high BF units at 1DPN. *p < 0.05. n ≅ 100 in each group.

The first evaluation of single unit data was on the change of SR after noise exposure. Figure 2 summarized the total units obtained in the 4 groups to show the distributions of both BF-threshold (A) and SR-threshold (B). It is seen that, the units with very low SR was reduced at 1PDN in association with the threshold elevation by noise at this time. A one-way ANOVA of rank (Kruskal-Wallis test) was performed respectively on SR changes for ANF units with low/high best frequency separated by a cutoff at 4 kHz. Significant changes in SR values was seen only in high-BF units (H_3_ = 13.314, p = 0.004). More importantly, the difference was only significant between the control units and those measured at 1DPN (Post-hoc test, Q = 3.176, P < 0.05, Fig. 3A). Figure 3B graphically shows the SR distribution across the groups. In addition, the change in SR distribution of ANFs was demonstrated by the change of ratio of low SR/high SR units (with a cut off of SR at 20 sp/sec). In the high-BF region, the ratio was 1.2 (55 versus 47 units) in the control group; it was reduced to 0.35 (18 versus 52 units) at 1DPN, due to the preferential loss of low-SR units. However, the ratio was largely but not completely recovered at 1WPN (0.75) and 1MPN (0.86).

**Figure 4:**
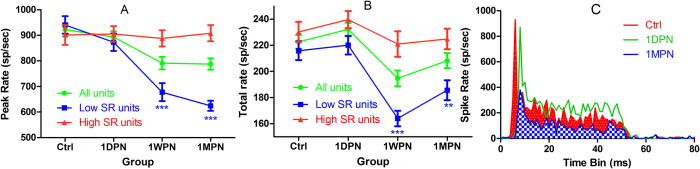
Noise-induced changes of driven spike rates. Significant effects were seen only in low-SR units and only appeared at 1WPN and 1MPN. (**A**) peak rate, (**B**) total rate. (**C**) Representative PSTHs from units obtained from the ctrl, 1DPN and 1MPN groups **p < 0.01, ***p < 0.001. The total units were 200 in each group and low-SR units were ~100.

The changes in signal encoding by ANFs were first evaluated in peri-stimulus histogram evoked by 50 ms tone burst. A later onset of coding changes in ANFs were evidenced in both intensity and temporal coding in the comparison of the PSTHs across groups. Examples of typical tone-burst PSTHs of low-SR units were presented in Fig. 4C, which shows the reduction in both peak and total spike rates in a unit obtained at 1MPN, but not at 1DPN. The elongation of peak latency was seen in the two noise-damaged units obtained both at 1DPN and 1MPN. Also, the reduction in peak/sustained ratio is visible in the PSTH of the 1MPN unit, as compared with the other two units. The exemplary data from 1WPN group was not shown because of the similarity with that of 1MPN data.

Two-way ANOVAs were performed to show the effects of noise (groups) and BF-regions, separately in units with low- and high-SR. In low-SR units, a significant effect of noise exposure was seen for the reduction of peak spike rate (F_3_ = 22.86, p < 0.001, Fig. 4A) and total spike rate (F3 = 14.03, p < 0.001, Fig. 4B). This effect of noise was not in high-SR units. There was no significant effect of BF-regions in both SR categories of ANF units. Post-hoc pairwise tests (Tukey method) against the control showed that the reduction in the driven spike rates (peak and sustained) was significant at 1WPN and 1MPN for both peak and sustained rates but not at 1DPN, revealing a later-onset reduction that was probably related with the repair of the synapses.

**Figure 5:**
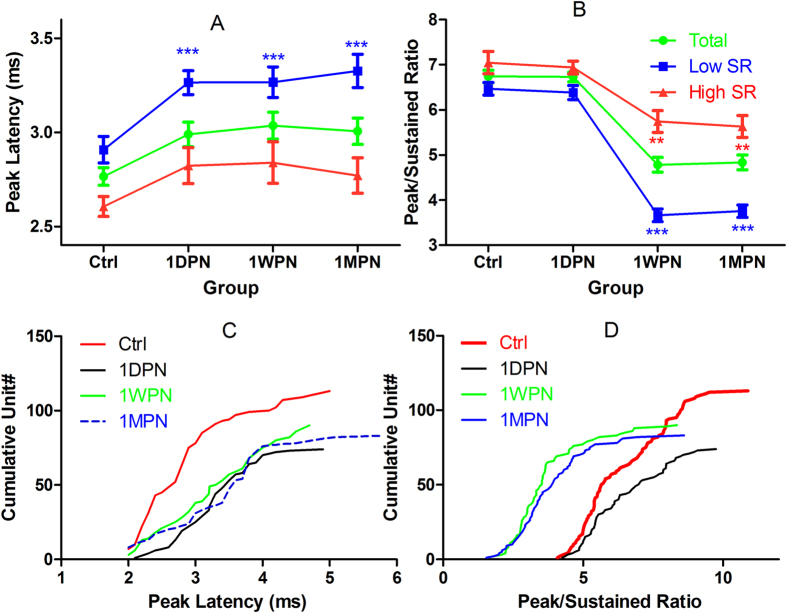
Noise induced changes on temporal responses of ANF units. (**A**) prolongation in peak latency, (**B**) reduction in peak-sustained spike ratio, (**C**) cumulative unit distribution of peak latency, and (**D**) cumulative unit distribution of peak/sustained ratio. **p < 0.01, ***p < 0.001 for the results of post-hoc pairwise comparison against the control.

**Figure 6:**
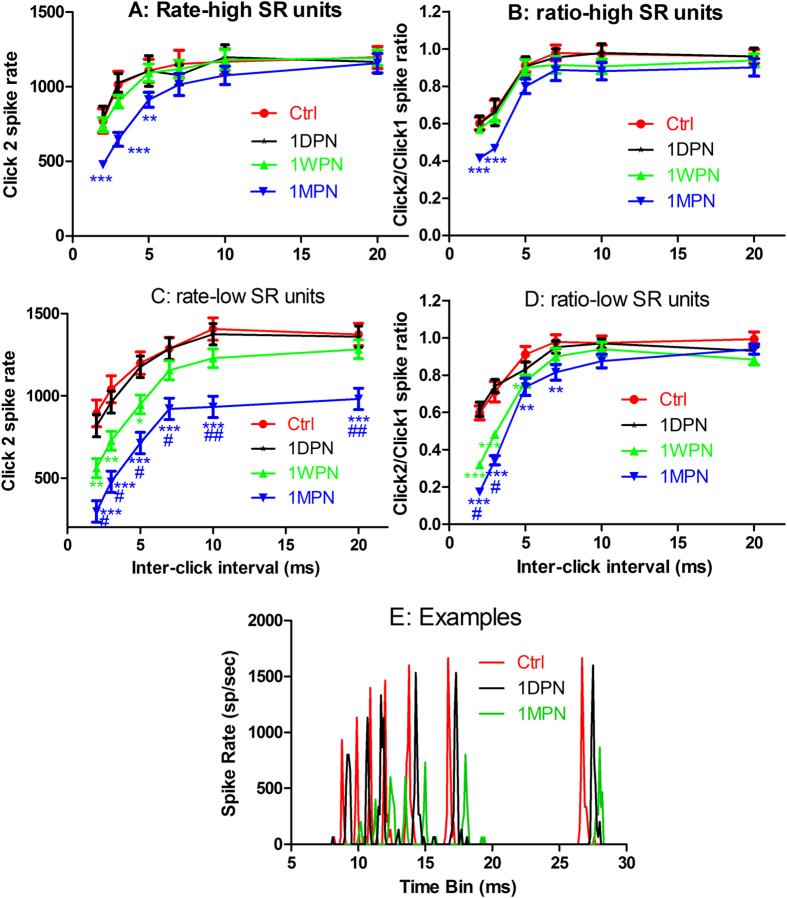
Impact of noise exposure on the recovery functions of click2 responses. Upper: high-SR units (**A,B**), middle: low-SR units (**C,D**), left: click2 spike rate-ICI functions; and right: click2/click1 ratio-ICI functions. (**E**) typical examples of PSTH for the spike rates evoked by the 2^nd^ clicks from 3 units obtained from the ctrl, 1DPN and 1MPN groups respectively. Two-way ANOVAs on the factors of groups (noise-treatment) and ICI showed significant effects of noise in both low- and high-SR units and in both the rate and the ratio. Asterisks indicated the significance in the post-hoc tests against the control within the factor of ICI (***p < 0.001, **p < 0.01). The significance was seen only at 1MPN in the high-SR units, but at both 1WPN and 1MPN in the low-SR units. The number symbols indicated the significance in the post-hoc pairwise tests within factor of IC and between 1MPN and 1WPN (^##^p < 0.01, ^#^p < 0.05).

The potential impact of the noise exposure on the temporal coding ability of ANFs were evaluated with (1) peak latency and (2) peak-to-sustained spike ratio in PSTH (Fig. 5A,B) as well as in the response recovery function in a paired-click regime (Fig. 6). Two-way ANOVAs (against the factor of noise groups and BF-regions) performed on peak latency showed a significant effect of noise in low-SR units (F3 = 7.061, p = 0.0001), but again not in the high-SR units (F3 = 1.239, p > 0.05) although the latency was increased in both types of units after noise. No significant effect of BF was seen. Post-hoc tests (Tukey method) for low-SR units show significant latency increases in peak latency at all post-noise time points (q = 4.624, P < 0.01; q = 4.921, p < 0.01; and q = 5.603, p < 0.001 for 1DPN, 1WPN and 1MPN respectively, Fig. 5A). Two-way ANOVAs also show significant effect of noise that resulted decreases in peak-sustained spike ratio, in both low- (F3 = 117.0, p < 0.001) and high-SR units (F3 = 12.01, p < 0.001). However, the post-hoc tests show only significant changes at 1WPN and 1MPN, but not at 1DPN (Fig. 5B). Figure 5C,D show the cumulative distribution of low-SR units for peal latency and peak/sustained ratio respectively.

In the paired-click regime, the ANF responses to the 2^nd^ click were measured as a function of inter click interval (ICI) varied from 2 to 20 ms (sFig. 3A). The spike rate to click 2 was suppressed by the preceding click 1 when the ICI was short; and was recovered with increasing ICI and approached to the rate evoked by click 1 for ICI >10 ms in the control samples (sFig. 3B). While there is a big difference in overall spike rate between low- and high-SR units (sFig. 3B), the recovery curves of click2/click1 spike rate ratio from the two categories of units were largely overlapped in normal control (sFig. 3C).

Figure 6 summarized the impact of noise on the recovery functions for both low- (lower panel) and high-SR units (upper panel) in the click2 spike rate (left column) and in ratio of click2-/click1-spike rates (right column). Overall, both the spike rate and the ratio were found to be lower in noise groups at short ICIs, suggesting a slower recovery of the click2 responses with increasing ICI. In high-SR units, there was no difference in the spike rate at the largest ICI, but in the low-SR units, there was a large reduction in the spike rate in every noise group even at the largest ICI, consistent with the reduction in the peak spike rates driven by tone bursts in the low-SR units. Examples of low-SR units were presented in Fig. 6E for units of control, 1DPN and 1MPN groups, in which an overall reduction in spike rate was seen in the unit of 1MPN group as compared to the control unit, plus a large reduction in spike rate at shorter ICIs. No big difference was see between the control and the 1DPN unit. Two-way ANOVAs were performed against the factors of noise groups and ICIs, respectively for both the click2 spike rate and the ratio in both low- and high-SR units. In each ANOVA, a significant effect of noise was seen. Further, pair-wise post-hoc tests verified at which ICIs the differences were significant. In general, the difference was larger at short ICIs, and more for low-SR units. Those results suggest that the noise exposure slow the recovery of ANF responses to the 2^nd^ clicks from the suppression of the proceeding click. This effect was larger in low-SR units. More importantly, this effect was only significant at the later time points (1WPN and 1MPN), but not showed off shortly after the noise exposure (1DPN).

### The changes in ribbon synapse morphology by TEM

**Figure 7:**
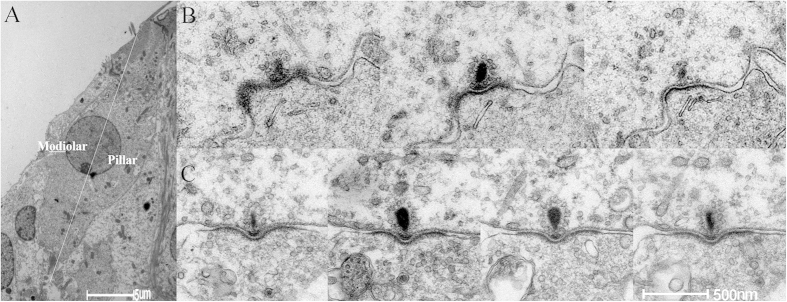
TEM images of a whole IHC (**A**) and serial sections of two ribbon synapses, one on the pillar side (**B**) and the other on the modiolar side (**C**). The larger ribbon volume at modiolar side is resulted from the larger cross-section area and/or the larger number of sections on which the ribbon is seen. The white lines show the measure of PSD angle.

IHCs were dissected in radial plane in order to verify the difference between the synapses on the modiolar side of IHCs and those on the pillar side. A central axis was drawn from the middle point of the head of IHC to the far end of the bottom in low profile images of IHCs to show the orientation (Fig. 7A). Totally 6 cochleae were examined in each group. Basilar membrane around 8 kHz region were sectioned for TEM (~70% or 12.5–14.5 mm from the apex, based upon the frequency-distance mapping of guinea pig cochlea^9^). In each cochlea, serial sections were taken over 10–20 μm after the nuclei of one IHC and three OHCs were seen in each slide. Totally, 800–1800 slides were collected from each group for observation. Table 1 summarized the number of synapses that were observed at each side across groups. It is shown that more synapses were seen at the modiolar side. More loss of synapses at this side was evident as the reduction of the modiolar/pillar ratio of synapse numbers at 1DPN, which was partially recovered later.

**Figure 8:**
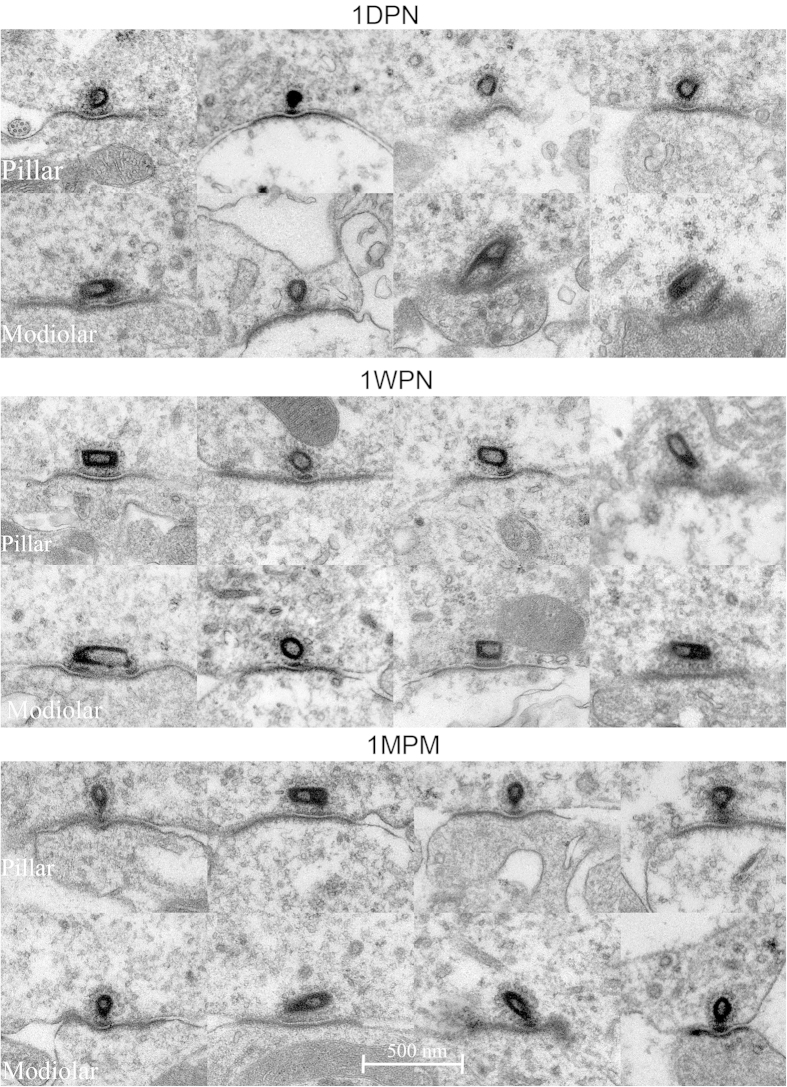
TEM images of ribbon synapses obtained at the 3 time points after the noise exposure. Swollen post-synaptic terminals were seed clearly in images at 1DPN. The presynaptic ribbons appeared to be larger, distorted and having a hollow center.

Figure 7B,C show the serial sections of two synapses in control sample at pillar and modiolar sides respectively. The ribbon at the modiolar side was relative larger as evident by larger cross areas and/or a larger number of ribbon-existing sections. In a limited number of synapses in each group (parentheses of Table 1), serial sections were obtained across the whole body of ribbons so that the volume of the ribbon was calculated as the sum of cross-section areas timed by the thickness (70 nm) of the section. In the control group, the volume of the ribbons at the modiolar side was found to be 3.51 ± 0.310 (10^6^ nm^3^). Figure 8 shows the representative images of ribbon synapses obtained at the 3 different time points after the noise exposure. Extremely swollen post-synaptic terminals, and larger-than-normal ribbons with distorted shape were seen in the images taken at 1DPN and 1WPN. Compared with the value of 2.47 ± 0.185 (10^6^ nm^3^) at the pillar side, the averaged ribbon volume at the modiolar side of IHCs was 42% larger than that at the pillar side. A slight increase in the ribbon volume was seen at the both sides in the groups measured after noise. However, a two-way ANOVA on ribbon volumes against the factor of noise treatments and sides across IHCs showed only a significant effect of sides (F1 = 26.456, p < 0.001) but not the effect of noise (F3 = 1.707, p = 0.167).

**Figure 9:**
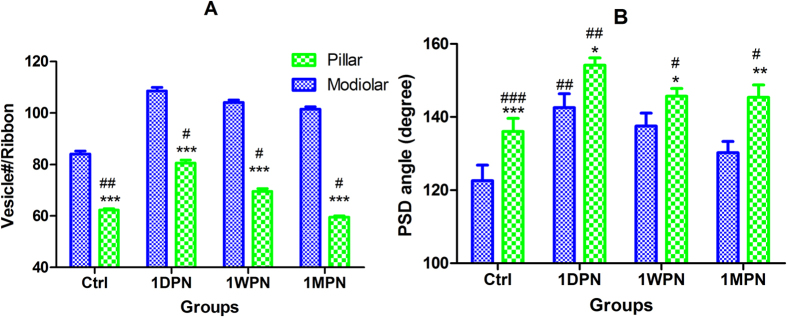
Comparisons of ribbon vesicles (**A**) and PSD angle (**B**) showing effect of sides (modiolar vs pillar) and noise groups. “#s” for the within side comparison against control, “*s” for the within group (time point) comparison between sides. ^###/^***p < 0.001, ^##/^**p < 0.01, ^#/^*p < 0.05.

In association with the post-noise increase in ribbon volume, two changes of ribbon structures attracted our attention. One is the hollowing of the ribbon (a low-density space inside the ribbons, Fig. 8). Such hollow spaces were seen also in the control in the control sample with a lower chance (18 out of 44 total ribbons counted at both sides, 40%), many more in the ribbons from noise groups (36/44 = 82% at 1DPN, 34/44 = 77% at 1WPN and 37/48 = 77%). We measured the hollow volume in a similar way as ribbon volume measurement. However, statistical analysis did not show a significance across the groups. The second change is the increase of the synaptic vesicles. A two-way ANOVA was performed and showed significant effects of both noise (groups) and side factors. In each group, the number of vesicles per ribbon was larger for ribbons at the modiolar sides. For example, this number was 84.0 ± 6.1 for ribbons at the modiolar side and 62.2 ± 2.3 for ribbons at the pillar side. The number of vesicles/ribbon was increased in the noise treated groups at both sides as shown in Fig. 9A. A two-way ANOVA showed significant effects of both noise (group) and side. Post-hoc tests (Holm-Sidak method) showed that within each groups, the number of vesicles was always significantly larger for ribbons at the modiolar side. At this side, the vesicle numbers of the three noise-groups were significantly larger than the control. There was no significant change at the pillar side.

The last change we observed was the angle of PSD. The significant effect of noise and side were also demonstrated by a two-way ANOVA, which showed that (1) the PSD angle in synapses at the pillar side were larger (PSD flatter) than in at the modiolar side within each group; (2) at both side, the PSD angle of each noise group was significantly larger than that of the control group (Fig. 9B). The length of PSD was measured but no difference was seen between sides and across groups.

## Discussion

The major findings of the present study include: (1) a large scale of synaptic repair after massive initial loss of ribbon synapse, (2) a transient change of SR distribution of ANFs due to initially disproportional loss of ribbon synapse innervating low-SR ANFs and later repair, (3) a delayed development of intensity and temporal coding deficits, manifested mainly in low-SR units in association with the re-establishment of disrupted synapses; (4) morphological changes of ribbon synapses, especially in presynaptic ribbons during the repair process.

Since the later onset of the deterioration in the signal coding abilities is accompanied with the synapse repair, the functional abnormalities are likely due to the larger contribution from those repaired units to the statistical values of the coding functions of the whole ANF population. This was manifested by the fact that the coding deficits were more significant in the low-SR units, which were disproportionally disrupted initially and repaired largely later. In the limited measures of ANF coding we observed, the low-SR ANFs show different functions from those high-SR units, including peak latency (Fig. 4A) and click-driven spike rate (sFig. 3A). However, the most striking finding of the present study is that the low-SR units become functionally poorer after their synapses with IHCs are repaired. Therefore, the problem is not simply the initial loss of the low-SR units by noise, but the functional changes in the repair. Further study is needed to verify if the deficits are persistent in longer time scale. Moreover, intensive investigation is needed to identify why the synapses with the low-SR ANFs are more vulnerable to noise, and why the repaired synapses are functionally abnormal.

The difficulties in auditory perception experienced by elderly are typically manifested as poorer temporal processing ability and low score of speech comprehension in noise background^10^,^11^,^12^. Such difficulties cannot be fully attributed with the degree of hearing loss or threshold elevations resulted from the pathologies in the peripheral auditory system^13^,^14^,^15^. Therefore, the temporal processing disorders and poorer perceptions in noise are often considered as the evidence of central presbycusis^12^,^16^,^17^,^18^,^19^,^20^,^21^. However, recent development in noise-induced silent damage to cochlea ribbon synapses have provided evidence to the peripheral origin for the auditory perception difficulty in noise^5^. The coding deficits developed after noise exposure reported in the present study actually provide and important mechanism or the reason for the auditory perception difficulty experienced by elderly. The details of the coding deficits reveals the nature of hidden hearing loss induced by noise exposures.

There is a debate about the fate of ribbon synapses damaged by noise. The noise-induced damage around ribbon synapses was investigated intensively 20 years ago by many researchers. In those early studies, morphological evidence was obtained for the partial repair of the synapses after initial damage^22^,^23^,^24^,^25^. Unfortunately, those observations were criticized as not being quantitative. New evidence showed that, at least in CBA mice, the damage is virtually irreversible^1^: the initial loss of ribbon counts in immunostaining was approximately 60% and the recovery that occurred within a week after the noise exposure was less than 10%. Interestingly, the permanent 50% loss of ribbon synapses was in consistent with the final loss of SGNs tested 2 years after the noise exposure^1^, further confirming the data of immunohistology on synapse counts. The irreversible loss of ribbon synapse were duplicated in a more recent reported from the same research group in young CBA mice^26^.

However, the data from guinea pigs appeared to show a different story. For example, a study in guinea pigs found a similar percentage loss of ribbon synapses 2 weeks after a noise exposure that did not cause PTS^2^. However, the final loss of SGNs, which were examined in a limited number of subjects 2 years after the noise exposure, was much less than the initial synapse loss examined two weeks after the noise exposure. Although the ribbon counts were not examined dynamically in this study, the discrepancy between the initial loss of synapses and the final death of SGNs suggests that some of the initially-disrupted synapses were re-established to allow the survival of SGNs. In two of our previous reports, we found a larger recovery of synapse counts that were verified by immunostaining against presynaptic CtBP2 (ribbon) and post synaptic density (PSD) in multi-time point observation^3^,^4^. This results was duplicated in the present study focusing on single unit activities. Comparing the data between mice and guinea pigs as summarized above, it seems that there may be a species difference in the ability to regenerate synapses after noise-induced damage. However, two recent studies using C57 mice (one from our lab) reported that the loss of ribbon synapses induced by non PTS-inducing noise is largely reversible^27^,^28^.

In a recent review, the possibility of the repair or synapse regeneration after noise was rejected and the recovery of CtBP2/PSD counts in guinea pig cochleae after noise exposure in our studies was attributed to up/down regulation of the synaptic protein^29^. However, a possibility of such repair has been supported by several lines of data. Firstly, plastic changes in the presynaptic component was seen in the existence of multiple presynaptic ribbons around one active zone^22^ and changes in the size and location of ribbons after the noise exposure^4^. Secondly, the repair was supported by the dynamic changes in CAP amplitude in the present report. Theoretically, the CAP amplitude measured at high sound levels is determined by (1) the number of ANFs that are functional and recruited, (2) the efficiency of individual auditory fibers (synapses), and (3) the synchrony across the ANF units. All of those may be changed by noise-induced ribbon-synapse damage. The disruption of synapses put the ANFs connected to them not functioning. Since the % CAP amplitude reduction at 1MPN was much smaller than the initial % loss of synapse counts at 1DPN, it is likely that some of the synapses are re-established; otherwise, the ANFs connected IHCs via the survived synapses must make high-than-normal contribution to CAP, which is unlikely. Thirdly, the re-establishment of synapses were supported by the single unit data in the present study. This was seen in the transient change of SR distribution, as well as in the later onset of changes in intensity and temporal coding behaviors.

Therefore, it is very likely that, at least in guinea pigs, the noise-induced damage at the ribbon synapses is partially repairable and the “selective” loss of low-SR units by noise is not permanent. Moreover, our data suggests that the noise does not purely damage the synapses connected to low-SR units. Synapses to high-SR units are also damaged. This is supported by the reduction of CAP amplitude, which is considered as weighted sum of the responses from median- and high-SR fibers^30^. However, a question remains unanswered is that whether the synapses are repaired or regenerated. In the present study, we did not address this issue.

In conclusion, the present study demonstrates that noise exposure without PTS creates massive damage on ribbon synapses. The initially disrupted synapses can be largely repaired. However, the deficits in intensity and temporal coding were developed with during the repair as the nature of synapopathy that was predicted. Further studies are needed to verify the mechanisms of the functional deficits, the long-term change of the deficits, as well as how they are related to the morphological changes in the repaired synapses.

## Materials and Methods

### Subjects and experimental outline

All the experiment procedures were performed under the regulation of a research protocol that was approved by the University Committee for Laboratory Animals of Southeast University, China (Permit number: SYXK 2011-0009). This protocol was developed in accordance with the National Institutes of Health guide for the care and use of laboratory animals (NIH publications N. 8023). Albino guinea pigs, irrespective of gender, with red eyes were obtained for this study from the Experimental Animal Service of Southeast University, a qualified provider for laboratory animals. Totally, 64 guinea pigs at age of 2–3 months were used and there were divided into control (Ctrl: n = 16) group and noise exposure group (n = 48), which were further divided into 3 subgroups according to the time of observation as 1 day, 1 week and 1 month post noise (1DPN, 1WPN and 1MPN, 16 in each subgroup).

Normal hearing sensitivity of every subject was verified in the test of auditory brainstem response (ABR). After fully recovery from the anesthesia for ABR test, the animals in noise group were given a noise exposure. At the designed time point after the noise exposure, single unit recording was performed after the examinations of distortion product optoacoustic emission (DPOAE) and compound action potential (CAP). After the functional observations, one cochlea from each animal was obtained for immunohistology observation of ribbon counts, and the other cochlea was used for transmission electronic microscope (TEM) observation.

### Noise exposure

The animals were exposed to a single dose of broadband noise at 105 dB SPL for 2 h when they were awake. They were unrestrained in a cage 60 cm below the horns of two loudspeakers; one was a low frequency woofer and the other was a high frequency tweeter. Electrical Gaussian noise was delivered to the speakers after power amplification. The acoustic spectrum of the sound was distributed mainly below 20 kHz as reported previously^3^. The frequency range for sound density 10 dB below the peak was between 3–14 kHz. The noise level was monitored using a ¼-inch microphone linked to a sound level meter (Microphone: 2520, SLM: 824, Larson Davis, Depew, NY, USA).

### ABR, DPOAE and CAP

For ABR, DPOAE and CAP recordings, the animals were anesthetized with pentobarbital (80 mg/kg, intraperitoneally), and the body temperature was maintained at 37.5–38 °C with a thermostatic heating pad. Three subdermal needle electrodes were used to record ABRs. The non-inverting electrode was inserted at the vertex in the middle point between the two eyes, and the reference and grounding electrodes were on the two earlobes. To record CAPs, a silver ball electrode, which was led to the non-inverting channel of the pre-amplifier, was placed on the round window membrane via a small hole penetrated on the bulla inferior and posterior to the external ear canal.

The hardware and software (BioSig and SigGen) from Tucker-Davis Technologies (TDT system III, Alachua, FL, USA) were used for stimulus generation and bio-signal acquisition. The acoustic stimuli were tone bursts of 10-ms duration with cos^2^ gating and a 0.5-ms rise/fall time. The stimulus were played through a broadband speaker (MF1 from TDT), which was placed 10 cm in front of the animal’s head. The evoked responses were amplified 20-fold with a TDT pre-amplifier (RA16PA) in which the signal was then digitized at a sampling rate of 25 kHz. The responses were averaged 1,000 times for ABR and 100 times for CAP. ABR thresholds were measured across the frequencies from 2 to 48 kHz with tone bursts at the rate of 21.1/s. At each frequency, the test was performed in a down sequence, starting from 90 dB SPL and then in 5-dB steps until the ABR response disappeared. The threshold was determined as the lowest level at which a repeatable wave III response could be obtained. CAP was recorded in response to clicks or tong bursts across the same range of SPLs as used in ABR recording.

DPOAE recording was performed also using BioSig and SigGen software. A earpiece was inserted into the ear canal of the guinea pigs for sound delivery and recording. The microphone output of the earpiece was connected to Etymotic microphone system (Etymotic 10B+, Etymotic Research, Elk Grove Village, IL, USA). The two primary tones were presented at a level of 65 dB SPL via two MF1 speakers, with the geometric means of the two tones spread from 1 to 16 kHz in octave step, and the frequency ratio (F1/F2) of 1.2. The DPOAE were collected and measured as the peak at 2f1-f2, the noise floor was measured in a 24 Hz band surrounding 2f1-f2.

### Single Unit Recording

The animal was continued to be maintained for body temperature and anesthesia after CAP and DPOAE recording. Pentobarbital was given via syringe pump at a rate of 10–20 mg/kg/hour. Trachea intubation was performed and respiration was maintained artificially during the single unit recording, which typically lasted 6–8 hours. The head of animal was clamped with a home-made device so that the head was bent down to expose the neck. The skin and the muscle tissue were removed from the back of the skull. The occipital bone was opened and the dura was resected with great care. The cerebellar lobes on one side was partially aspirated in order to expose the brainstem, which was slightly push away medially by two small cotton balls at the two sides of the outlet of the internal auditory meatus in order to expose the trunk of the auditory nerve.

Glass micropipettes were used as electrodes having impedances between 10 and 20 MΩ when filled with 1 M NaCl. The electrode was advanced remotely by micro-positioner (Model 2650, David KOPF Instruments, Tujunga, CA, USA) when a broadband noise burst of 30 ms was presented at a level around 75 dB SPL. The spikes was led from the electrode to the headstage of a microeletrode AC Amplifier (Model 1800, A-M Systems, Carlsborg, WA, USA). The output of the amplifier was sent to RZ5 of the TDT system for digitizing and further processing. The sound stimulation and recording was controlled by Brainware software via RZ6 of TDT hardware. Once a neuron was isolated in unit searching with noise burst, following items were recorded: (1) spontaneous spikes recorded for 30 seconds, (2) unit threshold estimated with the noise burst of different level, (3) best frequency (BF) estimated with iso-intensity-frequency curve by presenting 50-ms tone burst of different frequencies, (4) peristimulus histogram recorded to 50-ms tone burst at BF, and (5) responses to paired clicks of different inter click intervals (ICI, from 2 to 20 ms). In test (3), the stimulus at each frequency was repeated 50 times, while in test (4) and (5) the repetition was 150 times. In tests 3, 4 and 5, the sound level was 20 dB above the unit threshold. Due to the threshold elevation at 1DPN, the sound-level was in the range of 80–90 dB SPL, while at other time points, the sound level was widely distributed between 40–90 dB SPL. From test (4), peak latency, peak spike rate, adapted (sustained) spike rate as well as the peak/adapted spike rate ratio were measured or calculated. The peak spike rate (spikes/sec) was calculated using the spikes in the1 ms bin at the peak: the rate = spikes × 1000/150(sweep times). From test (5), the recovery of the response to the second click were measured as the function of ICI for both the spike rate against the second click or the ratio as spike rates evoked by the second divided by that evoked by the first click.

### Immunohistology

The major observation was the count of ribbons. In each group, the observation was performed successfully in 6–10 cochleae. After the end-point functional tests, the cochleae were harvested immediately and transferred to 4 °C phosphate-buffered saline (PBS), and then perfused rapidly with 4% paraformaldehyde in PBS buffer three times followed by brief post-fixation at 4 °C for 1 h and decalcification in 10% EDTA at room temperature for 6–10 h. The cochlea was then transferred to PBS, and the bone over the middle ear-facing portion of the cochlear spiral was removed using a fine forceps. After removing the tectorial membrane, the cochlea was permeabilized with 0.01% Triton X-100 in PBS for 30 min, incubated for 30 min in 5% goat serum in PBS and then incubated in the primary antibody (mouse anti-CtBP2 (C-terminal-binding protein 2) IgG1 from BD Biosciences, cat. # 612044, 1:200) overnight at 4 °C. The latter step was followed by treatment with secondary antibodies (goat anti mouse IgG1, 1:1000; Invitrogen A21124) for 2 h at room temperature. All of the antibodies used were diluted in 5% goat serum in PBS. After immunostaining, the basilar membrane was dissected into four pieces, mounted on microscope slides and cover slipped. To reduce the variability, and increase the reliability, of the results, control and experimental samples from the same time points were processed together under identical conditions.

Confocal images were acquired using a confocal laser-scanning microscope (Zeiss LSM 510 META) with ×100 oil-immersion objectives. Image stacks were then ported to image-processing software (Lsmix and ImageJ). The laser excitation power and microscope emission and detection settings were identical for all observations. Across the entire basilar membrane, immunoreactive puncta of CtBP2 were counted across a total of 17 regions in equal % distance step from the apex. In each region, the ribbon was counted from the IHCs observed in 2–3 microscopic fields, each typically with 9–11 IHCs. The total puncta for CtBP2 staining were divided by the total number of IHCs to obtain the average number of ribbons for each IHC.

### TEM observation

The cochleae were perfused for pre-fixation with immersing into 2.5% glutaraldehyde and then were immersed in the fixative overnight at 4 °C. Next, the basilar membrane was dissected under dissection microscope and post-fixed in 1% osmium solution (0.1 M PBS) for 1 hour. After that, the samples were subjected to dehydration with a graded series of ethanol concentrations from 50% to 100%, 15 min in each respectively. Then the samples were embedded in Eponate 12^TM^ kit (Ted Pella, 18010, Redding CA, USA) and polymerized at 60 °C for 48 hours. Semithin sections were collected and stained with toluidine blue (Urchem, 71041284, shanghai, China) for light microscope identification of targeted site. Ultra-thin sections for ribbon synapses were taken from HCs around 8 kHz region in the basilar membrane based upon the cochlear frequency map (Muller *et al*. 2005). The IHCs were cut in parallel with the long axis. After a ribbon was identified, a serial sections in thickness of 70 nm were taken across the whole ribbons in order to calculate the ribbon volume. The sections were collected on formvar-coated single-hole copper grids which allowed continuous views of the whole ribbon. The sections were stained with 2% aqueous uranyl acetate (Ted Pella, 19481) and lead citrate (Ted Pella, 19312). The samples were observed and images were taken with a TEM (FEI Tecnai Spirit (80 kV) at 1,200X, 3,300X and 9,900X magnifications respectively to observe entire IHC, and the spots of ribbon synapses. Totally 24 cochleae were observed, 6 in each group (Ctrl, 1PDN, 1WPN and 1MPN) and one cochlea from one animals. ImageJ software was used to measure the ribbon synapses in the ways similar to what were reported previously (Stamataki, 2006). The sum of cross-section areas and section interval were used to calculate the ribbon volume and the hollow volume inside. The volumes of ribbons and the hollow space inside were measured using 3D reconstruction technology in ImageJ, with a plug-in of TrakEM2 package provided by the Beijing Computational Science Research Center.

### Statistical analysis

All data are expressed as means ± standard error of the mean (SEM). Analysis of variation (ANOVA, one way or two way) was performed using SigmaPlot 11 software. P < 0.05 was used as the criterion of significant difference in all the tests.

## Additional Information

**How to cite this article**: Song, Q. *et al*. Coding deficits in hidden hearing loss induced by noise: the nature and impacts. *Sci. Rep*. **6**, 25200; doi: 10.1038/srep25200 (2016).

## Supplementary Material

Supplementary Information

## Figures and Tables

**Table 1:** The number of ribbon synapses at both side of IHCs: a comparison across groups.

Groups	Modiolar side	Pillar side	Ratio
Ctrl	111 (20)	45 (13)	2.47
1DPN	94 (24)	57 (20)	1.65
1WPN	69 (18)	33 (17)	2.09
1MPN	88 (32)	41 (16)	2.15

The numbers in parentheses are synapses from which serial sections were successfully obtained over the whole ribbon. From those synapse, the measurements of ribbon volume, number of vesicles, and PSD angle were performed.

## References

[b1] Kujawa S. G. & Liberman M. C. Adding insult to injury: cochlear nerve degeneration after “temporary” noise-induced hearing loss. J Neurosci 29, 14077–14085, doi: 29/45/14077 doi: 10.1523/JNEUROSCI.2845-09.2009 (2009). 19906956 PMC2812055

[b2] Lin H. W., Furman A. C., Kujawa S. G. & Liberman M. C. Primary neural degeneration in the Guinea pig cochlea after reversible noise-induced threshold shift. J Assoc Res Otolaryngol 12, 605–616, doi: 10.1007/s10162-011-0277-0 (2011). 21688060 PMC3173555

[b3] Liu L. . Silent damage of noise on cochlear afferent innervation in guinea pigs and the impact on temporal processing. PLos One 7, e49550, doi: 10.1371/journal.pone.0049550PONE-D-12-26008 (2012). 23185359 PMC3504112

[b4] Shi L. . Ribbon synapse plasticity in the cochleae of Guinea pigs after noise-induced silent damage. PLos One 8, e81566, doi: 10.1371/journal.pone.0081566PONE-D-13-30022 (2013). 24349090 PMC3857186

[b5] Plack C. J., Barker D. & Prendergast G. Perceptual consequences of “hidden” hearing loss. Trends in hearing 18, doi: 10.1177/2331216514550621 (2014). PMC4227662 25204468

[b6] Furman A. C., Kujawa S. G. & Liberman M. C. Noise-induced cochlear neuropathy is selective for fibers with low spontaneous rates. J Neurophysiol 110, 577–586, doi: 10.1152/jn.00164.2013 (2013). 23596328 PMC3742994

[b7] Young E. D. & Barta P. E. Rate responses of auditory nerve fibers to tones in noise near masked threshold. J Acoust Soc Am 79, 426–442 (1986). 3950195 10.1121/1.393530

[b8] Costalupes J. A. Representation of tones in noise in the responses of auditory nerve fibers in cats. I. Comparison with detection thresholds. J Neurosci 5, 3261–3269 (1985). 4078627 10.1523/JNEUROSCI.05-12-03261.1985 PMC6565232

[b9] Viberg A. & Canlon B. The guide to plotting a cochleogram. Hear Res 197, 1–10 (2004). 15504598 10.1016/j.heares.2004.04.016

[b10] Gordon-Salant S. Hearing loss and aging: new research findings and clinical implications. J Rehabil Res Dev 42, 9–24 (2005). 16470462 10.1682/jrrd.2005.01.0006

[b11] Divenyi P. L., Stark P. B. & Haupt K. M. Decline of speech understanding and auditory thresholds in the elderly. J Acoust Soc Am 118, 1089–1100 (2005). 16158663 10.1121/1.1953207 PMC1440523

[b12] Pichora-Fuller M. K. & Souza P. E. Effects of aging on auditory processing of speech. Int J Audiol 42 Suppl 2, 2S11–16 (2003). 12918623

[b13] Alain C., Zendel B. R., Hutka S. & Bidelman G. M. Turning down the noise: the benefit of musical training on the aging auditory brain. Hear Res 308, 162–173, doi: 10.1016/j.heares.2013.06.008 (2014). 23831039

[b14] Aydelott J., Leech R. & Crinion J. Normal adult aging and the contextual influences affecting speech and meaningful sound perception. Trends Amplif 14, 218–232, doi: 10.1177/1084713810393751 (2010). 21307006 PMC4111406

[b15] Boettcher F. A. Presbyacusis and the auditory brainstem response. J Speech Lang Hear Res 45, 1249–1261 (2002). 12546491 10.1044/1092-4388(2002/100)

[b16] Humes L. E. . Central presbycusis: a review and evaluation of the evidence. J Am Acad Audiol 23, 635–666, doi: 10.3766/jaaa.23.8.5 (2012). 22967738 PMC5898229

[b17] Glyde H., Hickson L., Cameron S. & Dillon H. Problems hearing in noise in older adults: a review of spatial processing disorder. Trends Amplif 15, 116–126, doi: 10.1177/1084713811424885 (2011). 22072599 PMC4040826

[b18] Walton J. P. Timing is everything: temporal processing deficits in the aged auditory brainstem. Hear Res 264, 63–69, doi: 10.1016/j.heares.2010.03.002 (2010). 20303402 PMC7045868

[b19] Lister J. J. & Roberts R. A. Effects of age and hearing loss on gap detection and the precedence effect: narrow-band stimuli. J Speech Lang Hear Res 48, 482–493 (2005). 15989406 10.1044/1092-4388(2005/033)

[b20] Schneider B. A. & Hamstra S. J. Gap detection thresholds as a function of tonal duration for younger and older listeners. J Acoust Soc Am 106, 371–380 (1999). 10420628 10.1121/1.427062

[b21] Walton J. P., Frisina R. D. & O’Neill W. E. Age-related alteration in processing of temporal sound features in the auditory midbrain of the CBA mouse. J Neurosci 18, 2764–2776 (1998). 9502833 10.1523/JNEUROSCI.18-07-02764.1998 PMC6793092

[b22] Ruel J. . Physiology, pharmacology and plasticity at the inner hair cell synaptic complex. Hear Res 227, 19–27, doi: S0378-5955(06)00247-4 doi: 10.1016/j.heares.2006.08.017 (2007). 17079104

[b23] Pujol R. & Puel J. L. Excitotoxicity, synaptic repair, and functional recovery in the mammalian cochlea: a review of recent findings. Ann N Y Acad Sci 884, 249–254 (1999). 10842598 10.1111/j.1749-6632.1999.tb08646.x

[b24] Puel J. L., d’Aldin C., Ruel J., Ladrech S. & Pujol R. Synaptic repair mechanisms responsible for functional recovery in various cochlear pathologies. Acta Otolaryngol 117, 214–218 (1997). 9105452 10.3109/00016489709117773

[b25] Pujol R., Puel J. L., Gervais d’Aldin C. & Eybalin M. Pathophysiology of the glutamatergic synapses in the cochlea. Acta Otolaryngol 113, 330–334 (1993). 8100108 10.3109/00016489309135819

[b26] Jensen J. B., Lysaght A. C., Liberman M. C., Qvortrup K. & Stankovic K. M. Immediate and delayed cochlear neuropathy after noise exposure in pubescent mice. PLos One 10, e0125160, doi: 10.1371/journal.pone.0125160 (2015). 25955832 PMC4425526

[b27] Shi L. . Noise induced reversible changes of cochlear ribbon synapses contribute to temporary hearing loss in mice. Acta Otolaryngol, 1–10, doi: 10.3109/00016489.2015.1061699 (2015). 26139555

[b28] Shi L. . Noise-induced damage to ribbon synapses without permanent threshold shifts in neonatal mice. Neuroscience 304, 368–377, doi: 10.1016/j.neuroscience.2015.07.066 (2015). 26232715

[b29] Kujawa S. G. & Liberman M. C. Synaptopathy in the noise-exposed and aging cochlea: Primary neural degeneration in acquired sensorineural hearing loss. Hear Res, doi: 10.1016/j.heares.2015.02.009 (2015). PMC4567542 25769437

[b30] Bourien J. . Contribution of auditory nerve fibers to compound action potential of the auditory nerve. J Neurophysiol 112, 1025–1039, doi: 10.1152/jn.00738.2013 (2014). 24848461

