# Supplementary material for: Coding deficits in hidden hearing loss induced by noise: the nature and impacts
Source: Sci Rep. 2016 Apr 27;6:25200. doi: 10.1038/srep25200 (PMC4846864; doi:10.1038/srep25200)
Supplement: Supplementary Information [file srep25200-s1.pdf]

Supplementary materials

Title: Coding deficits in hidden hearing loss induced by noise: the nature and impacts

Qiang Song, Pei Shen, Xiaowei Li, Lijuan Shi, Lijie Liu, Jiping Wang, Zhiping Yu, Kegan Stephen, Steve Aiken, Shankai Yin, Jian Wang

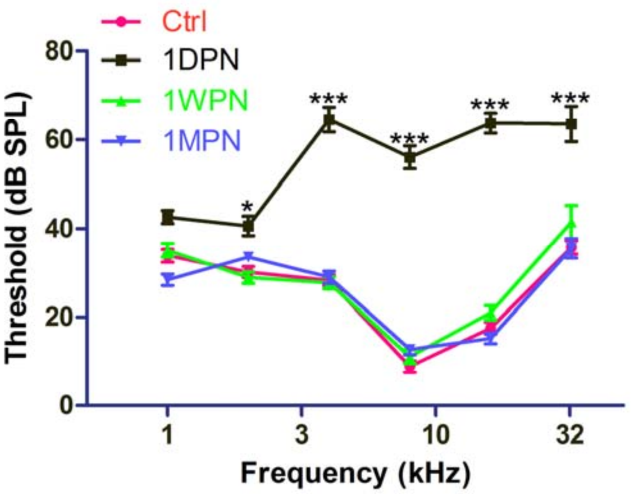

sFigure 1. The threshold-frequency curves (audiograms) obtained with the test of auditory brainstem responses. A two-way ANOVA shows a significant effect of noise (F_3=318.57, p<0.01). This was only due to the elevated thresholds at 1DPN as evident by the post-hoc pairwise compassion between 1DPN and the control data: q=34.943, p<0.001). *: p,0.05, ***: p<0.001.

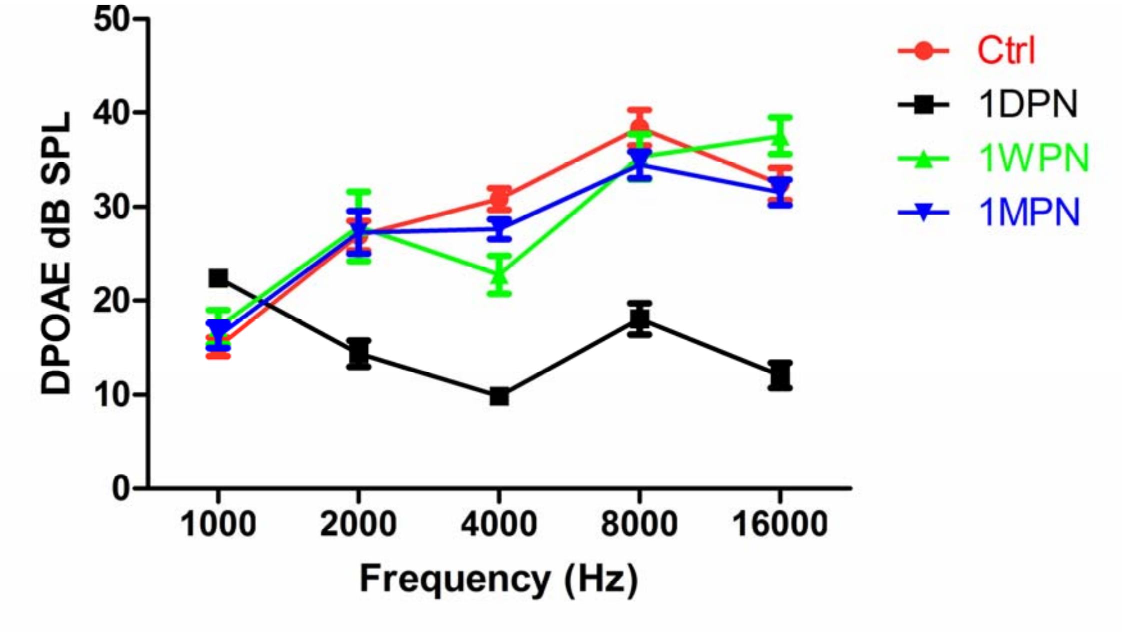

sFigure 2. DPOAE changes after the noise exposure, showing a transient depression of DPOAE amplitude at 1DPN and a total recovery tested later.

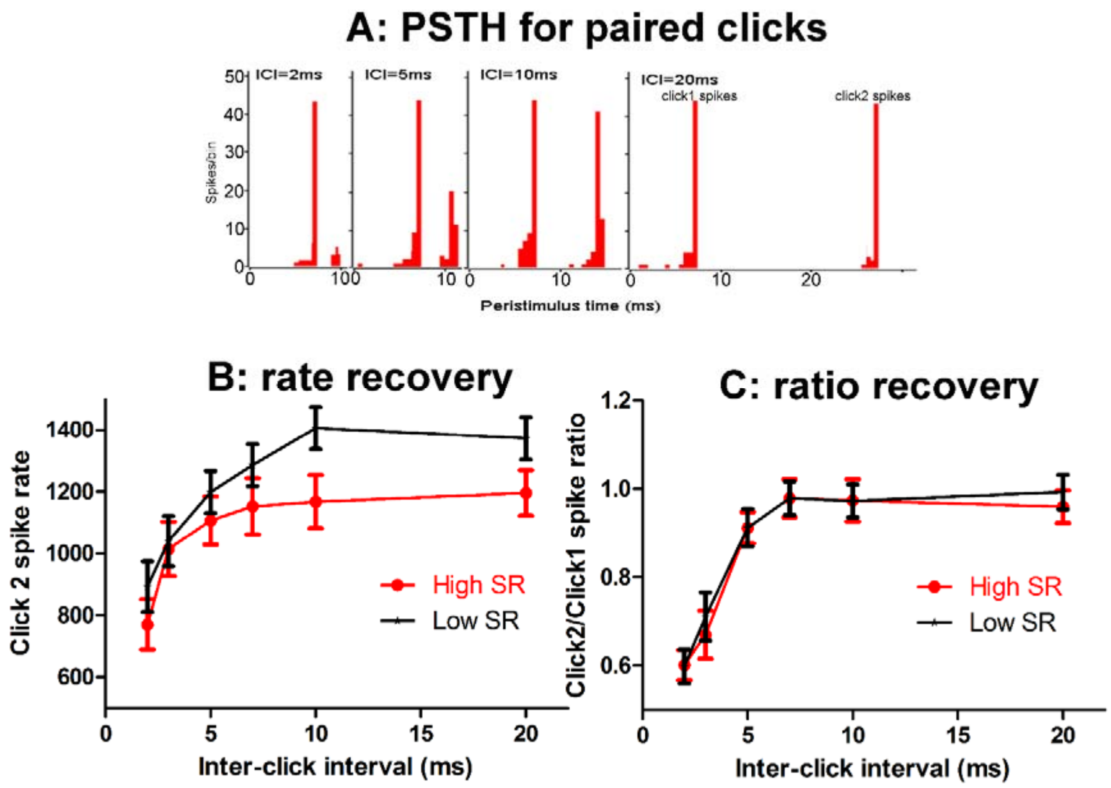

sFigure 3. Recovery functions of ANF responses to the second click in paired-click regime. A: Selected PSTHs of ANF responses to paired clicks showing the spike rate decrease with inter-click interval (ICI). B: the recovery of spike rates evoked by the second click as a function of ICI. C: the recovery of the ratio of 2nd click spike rate/first click spike rate.
